# Biochar and anionic polyacrylamide modulated soil hydraulic functions catalyze water saving, root development and yield of basmati rice

**DOI:** 10.3389/fpls.2025.1660325

**Published:** 2025-12-11

**Authors:** Peeyush Sharma, Vikas Abrol, Haziq Shabir, S. K. Gupta, N. K. Gupta, R. K. Samnotra, Subhash Chandra, Owais Bashir, Sheikh Amjid, Priti Singh, Abeer Hashem, Ajay Kumar, Elsayed Fathi Abd_Allah, Manish Kumar, Rubby Sandhu, Vinay Chembolu, Marcos Lado, Leena V Hublikar

**Affiliations:** 1 Division of Soil Science and Agricultural Chemistry, Sher-e-Kashmir University of Agricultural Sciences and Technology of Jammu, Jammu, India; 2 Directorate of Research, Sher-e-Kashmir University of Agricultural Sciences and Technology of Jammu, Jammu, India; 3 Division of Soil and Water Engineering, Sher-e-Kashmir University of Agricultural Sciences and Technology of Jammu, Jammu, India; 4 Division of Vegetables, Sher-e-Kashmir University of Agricultural Sciences and Technology of Jammu, Jammu, India; 5 Multidisciplinary Unit of Research on Translational Initiatives (MURTI) GITAM Deemed to be University and Department of Civil Engineering, GITAM School of Technology, Visakhapatnam, Andhra Pradesh, India; 6 Department of Botany and Microbiology, College of Science, King Saud University, Riyadh, Saudi Arabia; 7 Amity Institute of Biotechnology, Amity University, Noida, Uttar Pradesh, India; 8 Department of Plant Production, College of Food Science and Agriculture, King Saud University, Riyadh, Saudi Arabia; 9 Amity Institute of Environmental Sciences (AIES), Amity University Uttar Pradesh (AUUP), Noida, India; 10 Lovely Professional University, Jalandhar, Punjab, India; 11 Indian Institute of Technology, Jammu, India; 12 Department of Physics and Earth Science, University of A Coruna, A Coruña, Spain; 13 Department of Chemistry, KLE Technological University, Hubli, Karnataka, India

**Keywords:** biochar, polymer, soil hydraulic properties, system of rice intensification (SRI), root growth, water saving

## Abstract

**Introduction:**

Efficient water use while maintaining rice productivity remains a major challenge amid declining water resources. Biochar with large surface area and hydrophilic polymers, such as anionic polyacrylamide (PAM), can enhance soil moisture and nutrient retention. However, their combined effects on rice growth and soil hydro-physical properties have not been fully explored.

**Methods:**

This study evaluated the influence of biochar and PAM on soil properties, water retention, root growth, and rice performance under two establishment methods: the System of Rice Intensification (SRI) and Conventional Method (CM). A two-year field experiment was conducted on sandy clay loam soil using a split-plot design with SRI and CM as main plots and six soil amendment treatments (control, biochar, and polymer) as subplots, replicated thrice.

**Results and discussion:**

Results showed that SRI produced 15% higher grain yield while using 28.2% less irrigation water compared with CM. SRI also improved soil moisture (SM) content, infiltration rate (IR), hydraulic conductivity (HC), and water productivity (WP) by 6.6%, 64 5.4%, 9.0%, and 46.5%, respectively. Root length and weight densities under SRI were 1.31 and 1.62 times higher than under CM. The application of biochar (10 t ha⁻¹) and PAM (10 kg 66 ha⁻¹) significantly enhanced soil physical attributes, resulting in 1.22 and 1.27 times increases in grain yield and root length density, respectively, over the control. Integrating biochar and polymer with SRI reduced irrigation water use by 39.45% compared with CM. Overall, the combination of biochar (10 t ha⁻¹) and PAM (10 kg ha⁻¹) under SRI effectively improved soil hydro-physical properties, root development, water productivity, and rice yield in the sub-humid tropical Inceptisols of northern India.

## Graphical Abstract

**Figure:**
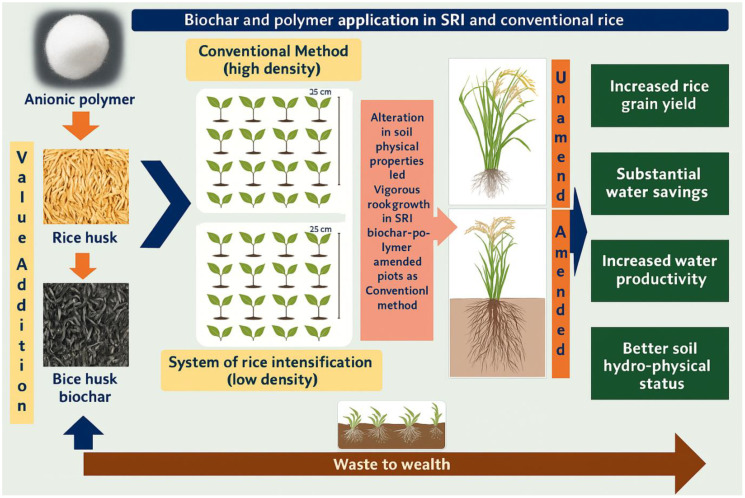

## Highlights

- Biochar and polymer amendments enhanced soil properties and crop yield.
- The SRI method achieved 15% higher rice yield with 39.5% less irrigation water.
- Combining SRI with biochar and polymer increased rice yield by 1.22-fold.
- Integrated SRI–biochar–polymer treatment reduced irrigation demand by 1.39-fold.
- Root length and weight densities improved by 1.31and 1.62-fold, respectively.

## 1 Introduction

Water scarcity and waste management in agriculture are primary bottlenecks in attaining the required growth in food productivity to sustain 9 billion people by 2050 (Deng et al., 2023). About 70% increase in food production is required to meet global food demands, while the water requirement for food and fiber production is projected to rise by 41.5% by 2050 (Mekonnen and Hoekstra, 2020).Agriculture alone consumes 72% of global freshwater, which is likely to escalate twice as fast as population growth (FAO, 2021). India is the world’s second-largest rice producer, followed by China, with 60% of its own consumption (Bandumula, 2018). According to a recent study, the primary rice producer country during the year 2020–2021 was China, which produced 148.30 million tonnes (Mt), followed by India (120.00 Mt), Bangladesh (35.30 Mt), Indonesia (34.90 Mt), and Vietnam (27.10 Mt) (Mohidem et al., 2022).

India contributes to 65% of the global rice production from only 20% of its cultivated land (Angha et al., 2024), wherein more than 90%of basmati rice production comes from the northwest states of India. Rice is a water-intensive staple food crop, consuming 34 – 43% of the global irrigation water and generating a considerablequantity of crop waste (Muthayya et al., 2014). On average, farmers use 3000–5000 liters of water to produce one kilogram of rice, whereas 2500 liters of water is enough to produce one kilogram of rice (Mandal et al., 2019). This highlights that 500–2500 liters of superfluous water is lost due to evapotranspiration, surface runoff, seepage, deep percolation, and the lack of adequate water-conserving techniques (Materu et al., 2018). Thus, it is imperative to develop appropriate water-conserving strategies that facilitate water retention, sustain rice yield and preserve ecological equilibrium in tropical and subtropical regions characterized by inadequate soil fertility and low water-holding capacity (Sharma et al., 2021; Saini et al., 2025).

The hydro-physical properties of soil significantly impact soil health by influencing the movement of water, root development and retention of nutrients (Blanco-Canqui et al., 2013; Abrol et al., 2024; Sharma et al., 2025b). Rice plants are traditionally cultivated by transplanting one-month-old seedlings in continuously flooded conditions. This method has several negative effects, including hindering root growth, contributing to greenhouse gas emissions, degrading soil structure (Dhanda et al., 2022; Ahmed et al., 2025), requiring a significant amount of water, and leading to low crop yields (Mandal et al., 2019; Mohapatra et al., 2023). The rice intensification (SRI) system is a novel rice cultivation technique that differs from conventional methods (Stoop et al., 2002; Mboyerwa et al., 2022). The key practices of SRI include transplanting younger plants in wider spacing, implementing alternate wetting and drying irrigation regimes, incorporating organic matter into the soil, and adopting judicious nutrient management. In addition, SRI involves soil management practices that include reduced water consumption, enhanced root development, improved soil properties through alternate wetting and drying conditions, and greatereconomic returns with higher crop yields compared to conventional methods (Thakur et al., 2013; Bagheri et al., 2021). Many researchers have also documented the adverse consequences of SRI on soil quality and crop yield (Pan et al., 2009; Sriphirom et al., 2019) and more pronounced root growth in flooded conditions compared to SRI (Suwarto et al., 2019).

Biochar is widely documented as a carbon-rich, low-cost soil amendment, adsorbent, and an alternative to agricultural waste management (Abrol et al., 2021; Kumar et al., 2022; Abhishek et al., 2022; Bolan et al., 2024; Shaheen et al., 2025; Sharma et al., 2025a). In addition, incorporating biochar into soil improves soil quality by increasing soil water content, promoting soil stability, stimulating microbial activity, and retaining nutrients, thus leading to increased crop productivity (Laird et al., 2010; Abrol et al., 2016; Rawat et al., 2019; Sharma et al., 2025b). Rice is cultivated in a major part of northern India under subtropical conditions that remain threatened by water scarcity due to uneven rainfall distribution, high temperature, and continuous water evaporation from flooded rice fields. Under these conditions, biochar amendment could be an effective strategy to combat these issues. Studies indicated that biochar application under water-scarce conditions in subtropical rice fields increased yield by 15-20% and enhanced water retention compared to untreated soils (Sharma et al., 2021). Furthermore, biochar aids carbon sequestration, increase crop yield and decrease toxic metal bioavailability due to its aromatic structure (Subedi et al., 2017; Kumar et al., 2018; Bolan et al., 2022; Ghani et al., 2024). Many researchers have reported an increase in root biomass with biochar application (Liu et al., 2021), while others have shown negative or no effect on root biomass (Zhu et al., 2020; Almaroai and Eissa, 2020).

Anionic polyacrylamide (PAM) is a high-molecular-weight polymer that can enhance aggregate stability, increase soil fertility, and prevent soil erosion (Sojka et al., 2007; Amiri et al., 2017; Kebede et al., 2020). Furthermore, PAM is an environmentally friendly material (Green et al., 2000), and it has been used as a soil amendment in agricultural field (Goebel et al., 2005; John et al., 2005). Many studies have shown that combining biochar with PAM improves soil structure, conserves water, and minimizes soil loss (Green et al., 2000; Abrol et al., 2016; Zhou et al., 2023; Deng et al., 2023).

The synergistic impact of biochar and polymers in System of SRIand flooded rice farming has seldom been examined by any previous studies. We posited that incorporating biochar and polymer as soil amendments will modify soil physical properties, thus affect crop performance and enhance water usage efficiency. This study aims to evaluate the effectiveness of biochar, polymer, and their combination on soil hydraulic parameters, root development dynamics, water retention, and rice yield under conventional and SRI system approaches.

## 2 Materials and methods

### 2.1 Location, climate, and soil

**Figure 1:**
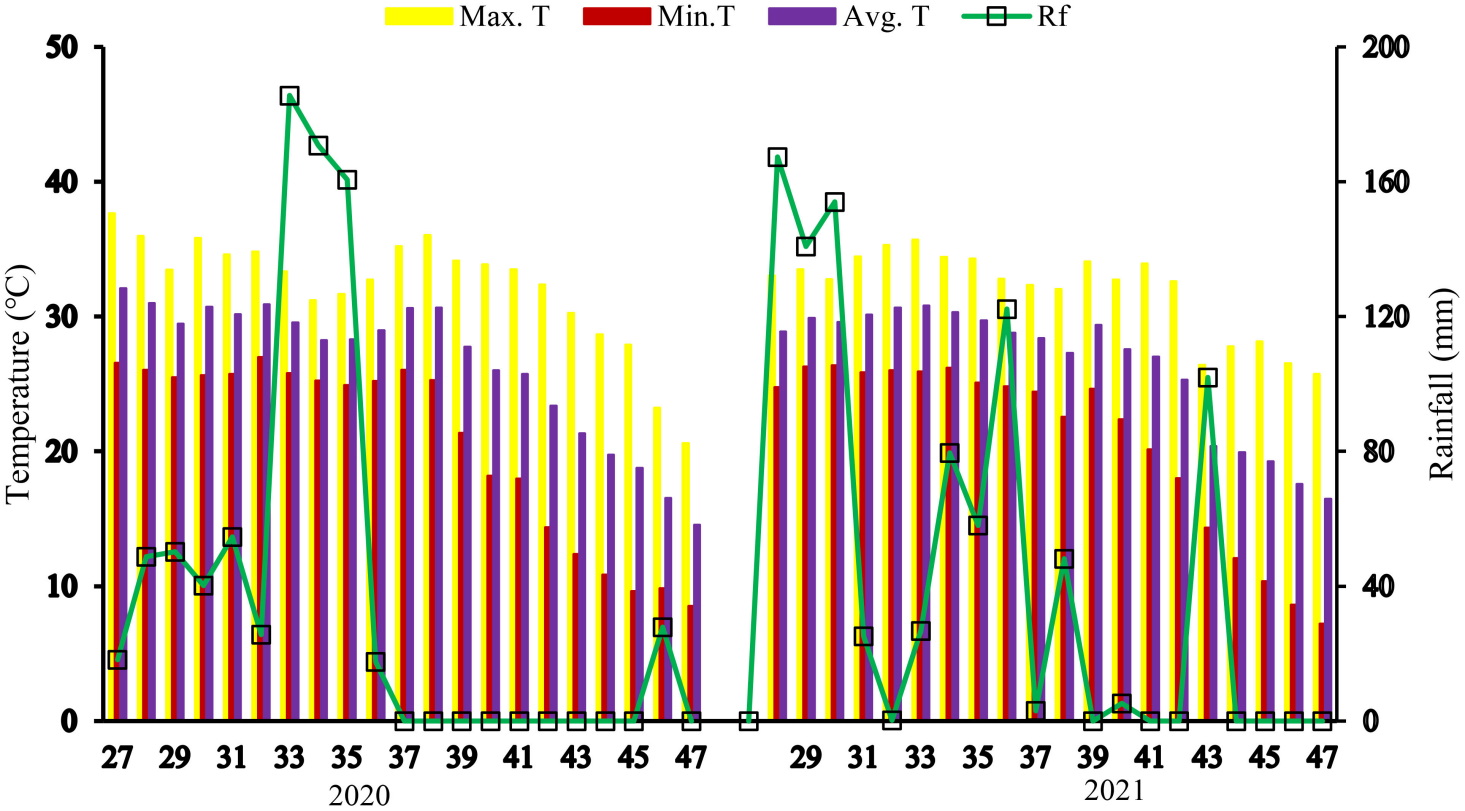
Average weekly temperature and rainfall during the Kharif season (July to Oct) in 2020 and 2021.

Field experiments were carried out on sandy clay loam soil during *Kharif* season(June-Oct) in 2020 and 2021 at Sher-e-Kashmir University of Agricultural Sciences and Technology of Jammu (32° 40′ N, 74° 58′ E). The study area is subhumid tropical, with an annual rainfall of 1077.8 mm. The total amount of rainfall obtained in the first and second years of the trial was 802mm and 934.5 mm, respectively. Weather data were collected from the Agrometeorological weather station in the university (**Figure 1**).Soil samples were collected from a depth of 0–15 cm at the time of rice harvesting and analyzed during both Kharif seasons across successive years. The soil pH was slightly alkaline (7.5) and contained 0.44% organic carbon, low in available nitrogen (205.24 kg ha^-1^), medium in phosphorus (12.63 kg ha^–1^), and high in potassium level (138.75 kg ha^–1^). The EC of soil was in safer limit for crop growth.

### 2.2 Experimental setup

The treatments comprised two rice establishment methods, viz, system of SRI and conventional flooded method (CM), in main plots along with six soil amendments (SA) treatments (control, sole and combined application of biochar and polymer) in subplots in a split-plot design replicated thrice. The treatment detail is presented in **Table 1**. The experimental plot size was 4 × 3m with two-meter buffer strips between the main plots and one meter between subplots to minimize adjacent movement of water. Rice was sown in the same plots for both years of study. Two seedlings per hill of Basmati 370 were transplanted at a spacing of 20×15 cm after attaining twenty-one days of growth in the conventional method, while in SRI, twelve-day-old seedlings per hill were planted, maintaining a spacing of 25×25cm. The recommended fertilizer (N 30:P_2_O_5_20:K_2_O10kgha^–1^) was applied in all the plots uniformly (100% RDF) using urea, diammonium phosphate, and muriate of potash in accordance with the package of practices. In the conventional method, 1/3 of the urea was applied during puddling and 2/3 was applied at tillering and before the panicle initiation stage. Under SRI practice, basal fertilizer application includes half the recommended nitrogen, and full dose of phosphorus and potassium, followed by a half dose of N (1/4^th^ at twenty days after transplanting and 1/4^th^ one week before panicle initiation).

**Table 1:** Summary of the treatments applied in the experiment.

Treatment	Biochar	Polyacrylamide
T_1_	0	0
T_2_	5 t ha^-1^	0
T_3_	0	10 kg ha^-1^
T_4_	5 t ha^-1^	10 kg ha^-1^
T_5_	10 t ha^-1^	0
T_6_	10 t ha^-1^	10 kg ha^-1^

T_1_, No amendment; T_2_, 5 tonnes biochar ha^-1^; T_3_, 10 kg polymerha^-1^; T_4_, 5 tonnes biochar ha^-1^ + 10 kg polymerha^-1^; T_5_, 10 tonnes biochar ha^-1^; T_6_, 10 tonnes biochar ha^-1^+ 10 kg polymerha^-1^.

EM, Establishment methods; SA, Soil amendments.

### 2.3 Irrigation details

To determine the irrigation requirement, the Penman method modified by FAO was taken as illustrated in Equation 1:

(1) $$ET_{c}=Kc\times ET_{0}$$

where,

ET_c_ = Crop Evapotranspiration (mmday^-1^)

K_c_ = Crop Coefficient

ET_0_= Reference crop evapotranspiration in mmday^-1^

K_c_ values of rice were calculated using FAO manual, and are presented in **Supplementary Table 1**. In CM, 1331 mm and 1096 mm irrigation depth was applied in 2020 and 2021, respectively to maintain a recommended irrigation depth of 70 mm depth. The subsequent irrigation was applied three days after the disappearance of ponded water. In the SRI, 1087 mm and 912 mm water was applied in 2020 and 2021, respectively, in irrigation depth of 25 mm after emergence of fine hair-like cracks in soil. The plots were kept at saturation from panicle initiation up to 15 days before harvesting. In both crop establishment methods, the plots were drained two weeks prior to harvesting.

Biochar was produced from rice husk by pyrolyzing it at 450°C for 2 hours in a slow pyrolysis rector and characterized for physicochemical properties as discussed in the earlier publication (Sharma et al., 2021).Before application, the biochar was crushed and sieved through 4 mm mesh manual sieve. An anionic granular polymer (A110; Cytec, Inc., North Andover, MA, USA) with a large molecular weight (12 × 10^6^ Da) was also used for supplementation. This polymer reportedly improves soil structural stability, as reported in previous studies (Ben-Hur et al., 1992; Levy et al., 1992; Yu et al., 2003). Both soil amendments, biochar and polymer were incorporated once into the soil before the transplantation ofthe rice crop as per treatment. Specifically, the biochar and polymer were applied into the soil in a sequence of six different treatment methods: T_1_: No amendment, T_2_: 5 tonnes biochar ha^-1^, T_3_: 10 kg polymerha^-1^, T_4_: 5 tonnes biochar ha^-1^ + 10 kg polymerha^-1^, T_5_: 10 tonnes biochar ha^-1^, T_6_: 10 tonnes biochar ha^-1^ + 10 kg polymerha^-1^.

### 2.4 Data collection and analysis

Plant root samples were extracted from each plot using a 8 cm diameter and 15 cm long core sampler in both seasons and washed over a 1 mm sieve. Root dry weight was measured after drying at 65°C for 24 hrs, and root length was calculated using the Tennant method (Tennant, 1975). Root morphological attributes, and including root diameter (RD), root volume (RV), root length density (RLD) (mmcm^–3^), and root weight density (RWD) (gcm^–3^), were computed using Equations 2 and 3:

(2) $$RLD=\frac{RL}{V}$$

(3) $$RWD=\frac{RW}{V}$$

where RL is the root length (cm), RW is the root weight (g), and V is the volume of the soil core.

At harvest, soil moisture (SM) was determined at a depth of 0–15 cm by digital moisture probe. The Decagon Minidisc infiltrometer was used to measure infiltration rate and hydraulic conductivity. The soil bulk density and penetration resistance were determined withcore (Blake and Hartge, 1986) and cone penetrometer methods, respectively. Water productivity (WP) and water saving (WS) were estimated using the Equations 4 and 5:

(4) $$Water\;productivity\;=\frac{Grain\;yield}{Total\;amount\;of\;water\;supplied}$$

(5) $$Water\;saving=\frac{x-y}{x}\times 100$$

where x is the amount of water applied in T_1_ in CM (m^3^ ha^-1^), and y is the amount of water applied (m^3^ ha^-1^).

Crop yield was measured for each subplot in a test area of 3m×3m, excluding border rows. At harvest, the number of effective tillers was determined for five tagged plants. The rice grain yield was determined at constant moisture content of grain of 14.5% and expressed in q ha^-1^.

### 2.5 Statistical analysis

The assumptions of normality and homoscedasticity were verified prior to conducting statistical analyses. The Shapiro–Wilk test was used to assess the normality of data distribution, while Levene’s test was applied to evaluate the homogeneity of variances. Accordingly, a two-way analysis of variance (ANOVA) was performed to evaluate soil and plant parameters obtained from the split-plot design comprising rice establishment methods (main plots) and soil amendment treatments (subplots) in accordance with Gomez and Gomez (1984). Mean values of three replicates were compared using Tukey’s Honestly Significant Difference (HSD) test at a 5% probability level (p ≤ 0.05). Linear regression analyses were also performed to examine the relationships between rice yield, root growth, and soil properties. Figures in the study were prepared using Origin software version 8.5.

## 3 Results

### 3.1 Biochar and polymer characterization

**Figure 2:**
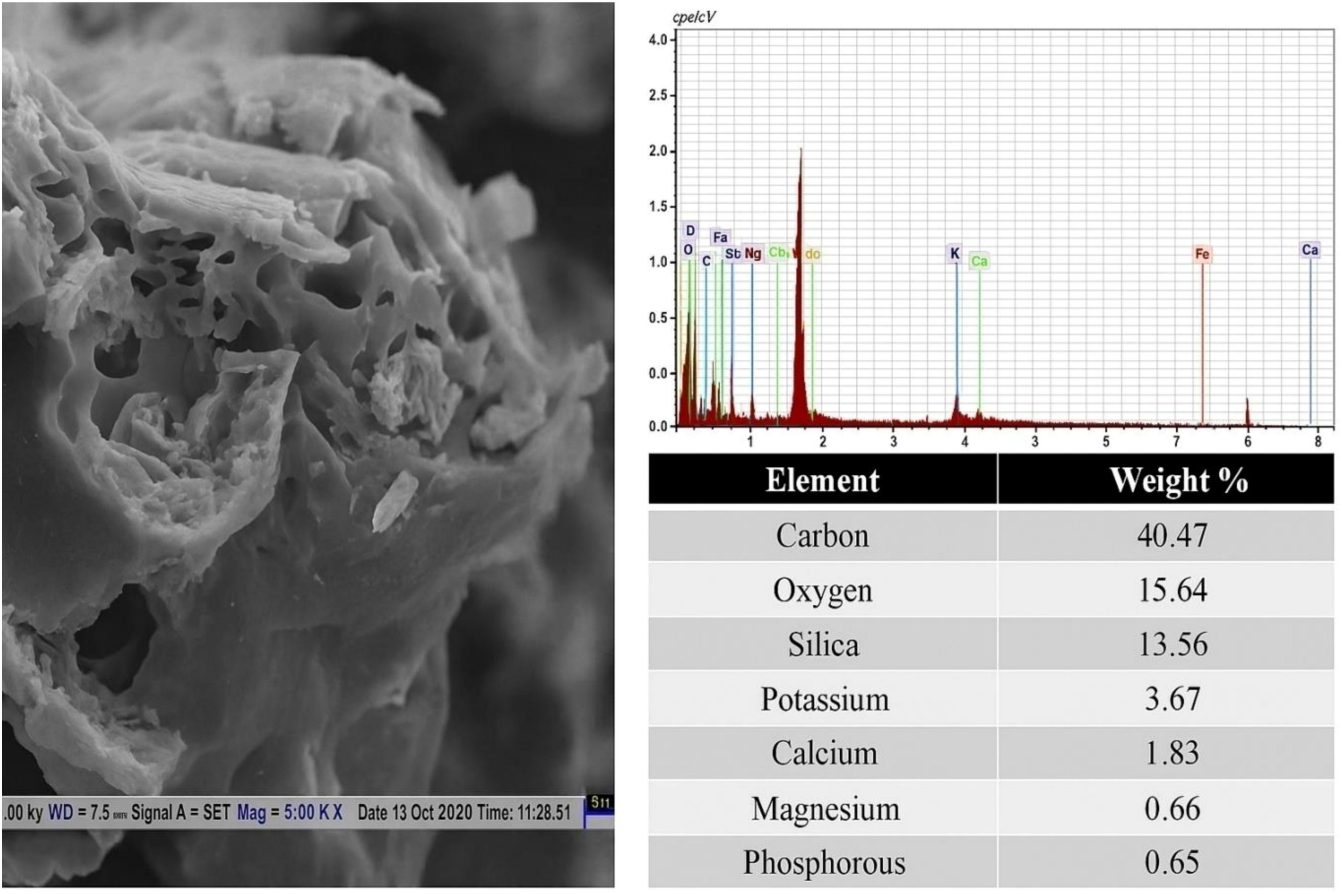
Morphological and EDX analysis of Rice husk-derived biochar.

The physicochemical properties of biochar derived from rice husk and PAM are shown in **Table 2**. Data indicates that biochar possesses a total carbon content of 45% and exhibits alkaline properties. Biochar had a substantial water retention capacity of 293%. The nutritional analysis indicated that the biochar has total phosphorus, nitrogen, and potassium levels of 0.17%, 0.69%, and 1.33%, respectively. This signifies that the biochar possesses a comparatively low phosphorus content, moderate nitrogen levels, and a high potassium concentration. **Figure 2** illustrates the Scanning Electron Microscope(SEM) and Energy-Dispersive X-ray(EDX) examination and showed that the biochar had a porous architecture characterized by both macro and micropores. The EDX analysis indicated that the biochar has approximately 40% carbon and 15.32% oxygen. Furthermore, it revealed the presence of silica oxide, potassium, calcium, magnesium, and phosphorus, which are essential soil components required for plant growth. The O/C ratio, an indication of aromaticity and biochar stability in soil, was 0.28 for the biochar. Our FTIR results indicated that the biochar blend of oxygenated and aromatic surface functional groups, making it a suitable soil amendment for improving crop yield due to increased water retention capacity, nutrient retention, and better stability in the soil (Sharma et al., 2025a; Saini et al., 2025).

**Table 2:** Physicochemical properties of rice husk biochar and anionic polyacrylamide (PAM).

Considerations	Biochar
Odour	Smoky
Colour	Black
EC (dSm^-1^)	0.42
pH	8.1
Total Carbon (%)	45.08
Waterholdingcapacity(%)	293
Total Phosphorous(%)	0.17
Total Nitrogen(%)	0.69
Total Potassium (%)	1.33

Chemical formula	**–[CH_2_–CH(CONH_2_)]_2_– with anionic groups**	
Charge	Anionic	
pH	7.67	
Density (g/cm^3^)	0.8	
Molecular weight	12 × 10^6^ Da	

**Figure 3:**
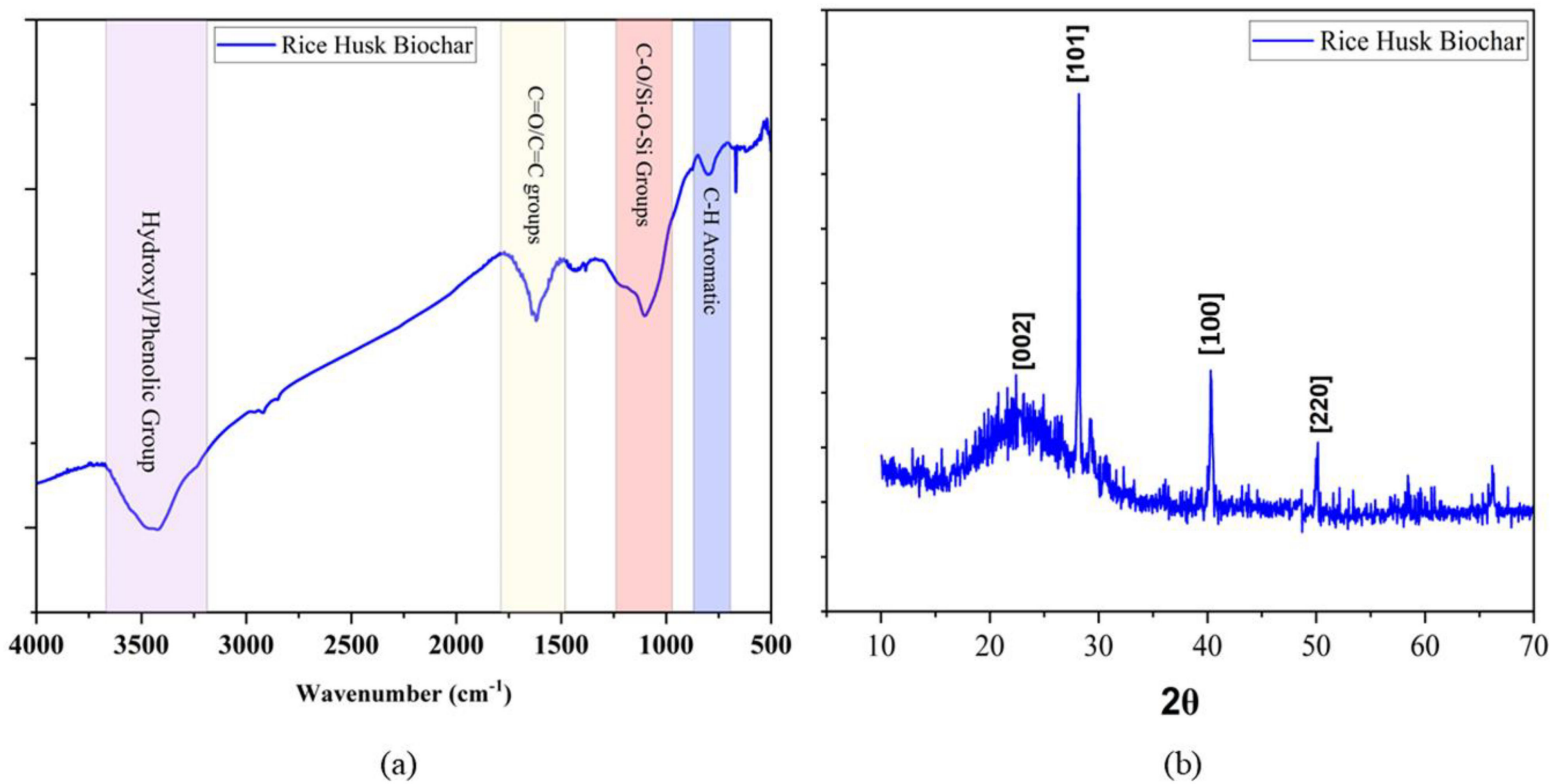
**(a)** FTIR analysis of Rice husk-derived biochar, **(b)** XRD analysis of Rice husk-derived biochar.

The Fourier Transform Infrared Spectroscopy(FTIR) and X-ray Diffraction (XRD) analysis are illustrated in **Figures 3a, b**. It can be observed from **Figure 3a** that the biochar contains several functional groups in the wavelength range of 4000–400 cm^–1^. Specifically, the vibrational frequency range of 3500–3300 cm^–1^ ascribed to the presence of hydroxyl and phenolic functional groups (Xiao et al., 2014*)*, 1680–1550 cm^–1^ linked to C=O/C=C aromatic carbon functional groups (Keiluweit et al., 2010*)*, 1090–1020 cm^–1^ linked to the presence of Si-O-Si/C-O (alcoholic and silica oxide) surface functional groups (Shen et al., 2017*)*, and 750–800 cm^–1^ can be ascribed to C-C aromatic functional groups (Chandra and Bhattacharya, 2019). The XRD analysis of the biochar (**Figure 3b**) showed the presence of various peaks at 23.20°, 28°, 40°, and 50°. The 23.20° and 28° peaks represent the amorphous carbon and silica oxide peaks (Chandra and Bhattacharya, 2019), respectively, with d-spacing of 0.382 nm and 0.316 nm, respectively. The (100) peak at 40° indicates the presence of graphitic carbon with a d-spacing of 0.223 nm. The last peak at 50° indicates the presence of a (220) plane corresponding to the silicon carbide with a d-spacing of 0.182 nm that might have formed during the pyrolysis process (Chandra and Bhattacharya, 2019).The anionic PAM polymer was directly purchased from the market and the basic properties were included with the packet as specified by the company. The basic physicochemical properties showed that the polymer possesses anionic surface characteristics, having a pH of 7.67 and a density of 0.8 g cm^-3^. The chemical formula (**Table 2**) indicates the presence of amine and carbonyl groups on the surface of the polymer.

### 3.2 Rice crop performance

**Figure 4:**
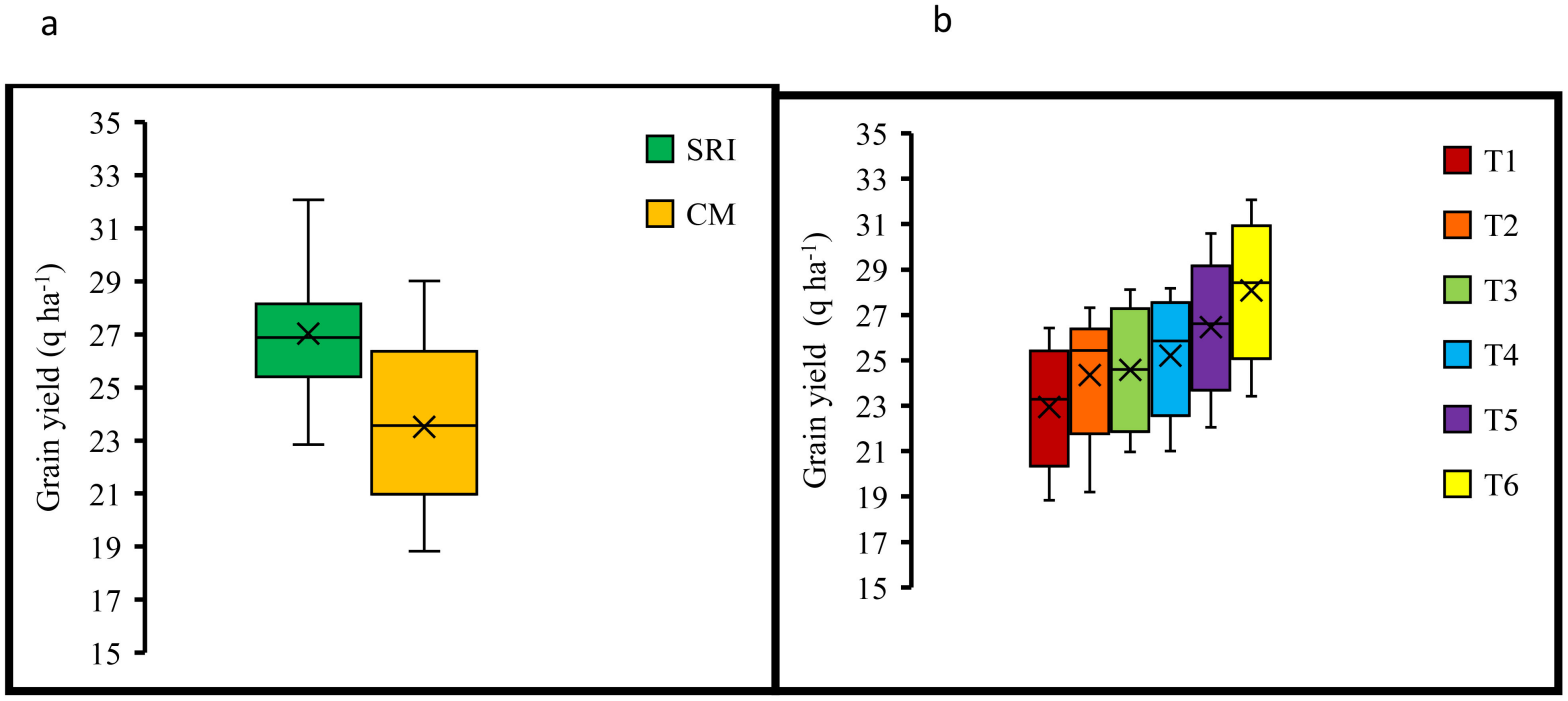
Box and whisker plot of grain yield with respect to **(a)** crop establishment methods, **(b)** soil amendments. Solid lines indicate median and the cross signs indicate mean. The box boundaries indicate the upper and lower quartiles and the whiskers indicate the 90th and 10th percentiles. Bars indicate standard error from mean P value< 0.05. SRI, System of rice intensification; CM, Conventional management; T1, No amendment (control); T2, 5 tonnes biochar ha^-1^; T3, 10 kg polymer ha^-1^; T4, 5 tonnes biochar ha^-1^ + 10 kg polymer ha^-1^; T5, 10 tonnes biochar ha^-1^; T6, 10 tonnes biochar ha^-1^+ 10 kg polymer ha^-1^.

Rice establishment methods (EM) significantly affected the rice yield during the two years of experimentation (**Table 3**). SRI obtained significantly higher rice yield than the conventional method in both years, and the maximum average grain yield was observed in SRI (27.02 q ha^-1^), which was significantly higher than that of the conventional method. Among soil amendment (SA), a substantial increase in grain yield 21.8% and 23%was observed with the application of 10 tonnesbiochar ha^-1^ + 10 kg polymer ha^-1^(T_6_) over 100% RDF (T_1_) for the years 2020 and 2021, respectively. The highest average grain yield (28.08 q ha^-1^) was recorded in the T_6_ treatment, and the lowest was noticed in the control (22.95 q ha^-1^). The interaction effect of the planting method and soil amendments was not significant in the first year; however, it was significant in the second year, and treatment T6 × SRI had the highest mean yield (22%) compared to the control. The lowest yield was recorded in T_1_ (no biochar) ×CM. The magnitude of the increase in grain yield in SRI was 16.7% compared to the conventional method. In contrast,the co-application of biochar and polymer (T_6_) showed a 22.4% increase over the control (**Figure 4**).

**Table 3:** Effect of establishment methods and soil amendments on grain yield and effective tillers density.

Treatments	Grain yield (q ha^-1^)							Effective tillers (number of tillers m^-2^)					Mean
	2020		Mean	2021		Mean		2020		Mean	2021		
	Establishment methods			Establishment methods				Establishment methods			Establishment methods		
	SRI*	CM		SRI	CM			SRI	CM		SRI	CM	
T_1_	26.42	23.72	25.07^c^	22.84	18.83	20.84^d^		194.07	187.69	190.88^d^	213.16	195.73	204.44^b^
T_2_	27.33	24.82	26.08^bc^	26.03	19.21	22.62^c^		197.71	191.13	194.42^c^	212.73	195.29	204.01^b^
T_3_	28.12	25.88	27.00^bc^	23.33	20.97	22.15^c^		196.49	190.78	193.64^c^	216.35	195.14	205.75^b^
T_4_	28.17	26.51	27.34^bc^	25.18	21.00	23.09^b^		201.95	195.25	198.60^b^	223.61	185.37	204.49^b^
T_5_	30.58	26.81	28.70^ab^	26.42	22.05	24.23^b^		205.03	197.48	201.25^a^	248.95	214.66	231.81^a^
T_6_	32.07	29.01	30.54^a^	27.83	23.42	25.63^a^		206.09	200.20	203.14^a^	252.48	212.67	232.57^a^
Mean	28.78^a^	26.13^b^		25.27^a^	20.91^b^			200.22^a^	193.75^b^		227.88^a^	199.81^b^	
EM SA EM×SA SA×EM	**			**				**			**		
	**			**				**			**		
	ns			**				ns			**		
	Ns			**				ns			**		

*SRI, system of rice intensification; CM, conventional management; EM, establishment method, SA, soil amendment; EM×SA, Interaction of establishment methods at same level of soil amendments; SA×EM, Interaction of soil amendments at same level of establishment methods. T_1_, No amendment; T_2_, 5 tonnes biochar ha^-1^; T_3_, 10 kg polymerha^-1^; T_4_, 5 tonnes biochar ha^-1^+ 10 kg polymerha^-1^; T_5_, 10 tonnes biochar ha^-1^; T_6_: 10 tonnes biochar ha^-1^+ 10 kg polymerha^-1^.

Different letters within the same column indicate significant differences between treatments, as determined by Tukey’s test with *p<* 0.05.

ns and **denotes not significant and significant differences at the 5% level, respectively.

Similarly, planting methods and soil amendments (SA)significantly impacted tiller density in both years (**Table 3**). According to the results, the SRI system significantly enhanced the number of tillers compared to CM throughout the experiment. The mean tillers m^-2^ increased by 8.8% under SRI compared to CM. Among the soil amendments, the polymer and biochar (T6) combination produced more tillers m^–2^ than the other treatments. Across years, a significant interaction between planting method and amendments was observed in SRI × T_6,_ resulting in an18.4% increase in tillers m^-2^ over the control in the second year.

### 3.3 Soil hydro-physical properties

The experimental data showed that EM and SA had a significant impact on SM, HC, and IR (**Table 4**). However, neither the EM nor the SA altered bulk density and penetration resistance (PR) over the two years, and only significant PR was observed in the second year (**Table 5**). No significant differences in PR, HC, and IR were observed between SRI and the CM in the first year; however, it was significant in the second year. SRI significantly increased the average SM (6.6%), HC (3.08%), and IR (5.3%) compared to CM. The T_2_, T_3_, T_4_, T_5_ and T_6_ treatments increased soil moisture by 4.7%, 3.8%, 7.9%, 13.1% and 21.3%, respectively, compared to control. The combination of biochar and polymer (T_6_) recorded the highest increase in HC and IR, with an increase of 1.33 and 1.65 times, respectively. Gradual reductions in PR were recorded in plots amended with biochar and polymer compared to those in the control. EM and SM had significant interaction effects in terms of HC and IR in the second year. The SRI × T_6_ treatment resulted in the highest mean values of SM (19.14%), HC (1.5 cm h^-1^), and IR (0.47 cm h^-1^). Also, EM and SA had no interaction effect on penetration resistance in either year.

**Table 4:** Effect of establishment methods and soil amendments on soil moisture, infiltration rate, and hydraulic conductivity. .

Treatments	Soil moisture (%)						Infiltration rate (cm hr^-1^)							Hydraulic conductivity (cm hr^-1^)					
	2020			Mean	2021		Mean	2020		Mean	2021		Mean	2020		Mean	2021		Mean
	Establishment methods				Establishment methods			Establishment methods			Establishment methods			Establishment methods			Establishment methods		
	SRI*		CM		SRI	CM		SRI	CM		SRI	CM		SRI	CM		SRI	CM	
T_1_	13.97		12.81	13.39^c^	16.94	16.61	16.77^e^	0.31	0.28	0.30^c^	0.27	0.26	0.27^d^	0.35	0.33	0.34^b^	0.33	0.32	0.33^e^
T_2_	14.20		13.25	13.73^bc^	18.05	17.67	17.86^c^	0.37	0.32	0.34^b^	0.37	0.38	0.38^c^	0.38	0.35	0.36^b^	0.40	0.32	0.36^d^
T_3_	14.60		13.84	14.22^bc^	17.67	16.55	17.11^d^	0.37	0.34	0.36^b^	0.40	0.36	0.38^c^	0.41	0.38	0.39^ab^	0.42	0.35	0.38^d^
T_4_	14.98		14.01	14.50^bc^	18.70	17.40	18.05^c^	0.38	0.37	0.37^b^	0.44	0.41	0.43^b^	0.43	0.39	0.41^a^	0.42	0.41	0.42^c^
T_5_	15.23		14.37	14.80^b^	19.99	18.67	19.33^b^	0.43	0.45	0.44^a^	0.47	0.46	0.47^a^	0.46	0.41	0.44^a^	0.47	0.48	0.47^b^
T_6_	17.43		15.55	16.49^a^	20.85	19.37	20.11^a^	0.47	0.45	0.46^a^	0.48	0.47	0.48^b^	0.47	0.42	0.45^a^	0.54	0.52	0.53^a^
Mean	15.07^a^		13.97^b^		18.70^a^	17.71^b^		0.39^a^	0.37^b^		0.41^a^	0.39^b^		0.42	0.38		0.43^a^	0.40^b^	
		**			**			ns			**			ns			**		
		**			**			ns			**			ns			**		
		ns			**			ns			ns			ns			**		
		ns			**			ns			ns			ns			**		

*SRI, system of rice intensification; CM, conventional management; EM, establishment method, SA, soil amendment; EM×SA, Interaction of establishment methods at same level of soil amendments; SA×EM, Interaction of soil amendments at same level of establishment methods. T_1_, No amendment; T_2_, 5 tonnes biochar ha^-1^; T_3_, 10 kg polymerha^-1^; T_4_, 5 tonnes biochar ha^-1^ + 10 kg polymerha^-1^; T_5_, 10 tonnes biochar ha^-1^; T_6_, 10 tonnes biochar ha^-1^ + 10 kg polymerha^-1^.

Different letters within the same column indicate significant differences between treatments, as determined by Tukey’s test with *p<* 0.05.

ns and **denotes not significant and significant differences at the 5% level, respectively.

**Table 5:** Effect of establishment methods and soil amendments on penetration resistance, infiltration rate, and hydraulic conductivity.

Treatments	Penetration resistance (M Pa)					Bulk density (g cm^-3^)						
	2020		Mean	2021		Mean	2020		Mean	2021		Mean
	Establishment methods			Establishment methods			Establishment methods			Establishment methods		
	SRI*	CM		SRI	CM		SRI	CM		SRI	CM	
T_1_	1.07	1.12	1.09	0.95	0.98	0.97^a^	1.40	1.41	1.40	1.42	1.42	1.42
T_2_	1.06	1.07	1.07	0.92	0.93	0.92^b^	1.40	1.41	1.40	1.41	1.42	1.41
T_3_	1.06	1.03	1.05	0.96	0.97	0.97^a^	1.39	1.40	1.40	1.41	1.42	1.42
T_4_	1.03	1.06	1.05	0.91	0.89	0.90b^c^	1.40	1.40	1.40	1.41	1.42	1.41
T_5_	1.04	1.04	1.04	0.84	0.88	0.86^cd^	1.39	1.39	1.39	1.41	1.41	1.41
T_6_	1.04	1.05	1.05	0.83	0.85	0.84^d^	1.39	1.40	1.39	1.40	1.41	1.41
Mean	1.05	1.06		0.90	0.92		1.39	1.40		1.41	1.42	
EM SA EM×SA SA×EM	ns			ns			ns	ns		ns		
	ns			**			ns	ns		ns		
	ns		ns	ns		ns	ns	ns		ns		
	ns		ns	ns		ns	ns	ns		ns		

*SRI, system of rice intensification; CM, conventional management; EM, establishment method, SA, soil amendment; EM×SA, Interaction of establishment methods at same level of soil amendments; SA×EM, Interaction of soil amendments at same level of establishment methods. T_1_, No amendment; T_2_, 5 tonnes biochar ha^-1^; T_3_, 10 kg polymerha^-1^; T_4_, 5 tonnes biochar ha^-1^ + 10 kg polymerha^-1^; T_5_, 10 tonnes biochar ha^-1^; T_6_, 10 tonnes biochar ha^-1^+ 10 kg polymerha^-1^.

Different letters within the same column indicate significant differences between treatments, as determined by Tukey’s test with *p<* 0.05.

ns and **denotes not significant and significant differences at the 5% level, respectively.

### 3.4 Root development

In both years, conventional and SRI practices and SA exerted substantial impacts on the RLD and RWD of basmati rice (**Table 6**). Irrespective of the soil amendments, the SRI yielded significantly higher average RLD (0.0308 mm cm^-3^) and RWD (0.0195 g cm^-3^) in both years than the conventional method. Under SRI, RLD and RWD increased by 31.1% and 63.8% in the first year and by 31.04% and 61.6% in the second year, respectively, compared to the conventional method. Furthermore, adding biochar and polymer increased the RLD and RWD compared to the control. Among the SA treatments, the highest RLD and RWD values were recorded for the combined application of 10 tonnes biochar ha^-1^ and 10 kg PAM ha^-1^ (T_6_) (0.0318 mm cm^-3^ and 0.021 g cm^-3^, and 0.0313 mm cm^-3^ and 0.0221 g cm^-3^ in 2020 and 2021, respectively), and the lowest RLD and RWD values were obtained in the control (0.0225 mm cm^-3^ and 0.0098 g cm^-3^, and 0.0272 mm cm^-3^and 0.0108 g cm^-3^ in 2020 and 2021, respectively). The magnitude of the increase in RLD and RWD over two years was 1.27 to 2.10 times greater than that in T_6_ treatment over RDF. In general, increasing doses of biochar and polymer markedly increased the RLD and RWD. In both years, the interaction effect of EM and SA was significant for RWD. However, for RLD, this interaction was only significant in 2021, and the greatest mean RLD (0.0343 mm cm^-3^) and RWD (0.02703 gm cm^-3^) were obtained for T_6_×SRI, while the lowest values were recorded for T_1_× CM.

**Table 6:** Effect of establishment methods and soil amendments on root weight density (RWD) and root length density (RLD) of Rice.

Treatments	Root weight density (g cm^-3^)						Root length density (mm cm^-3^)					
	2020		Mean	2021		Mean	2020		Mean	2021		Mean
	Establishment methods			Establishment methods			Establishment methods			Establishment methods		
	SRI*	CM		SRI	CM		SRI	CM		SRI	CM	
T_1_	0.0113	0.0083	0.0098^e^	0.0120	0.0095	0.0107^e^	0.0245	0.0205	0.0225^c^	0.0325	0.0219	0.0272^d^
T_2_	0.0122	0.0090	0.0106^d^	0.0176	0.0111	0.0144^d^	0.0255	0.0211	0.0233^bc^	0.0296	0.0251	0.0274^d^
T_3_	0.0164	0.0100	0.0132^c^	0.0180	0.0102	0.0141^d^	0.0269	0.0212	0.0241^bc^	0.0332	0.0228	0.0280^c^
T_4_	0.0206	0.0122	0.0164^b^	0.0210	0.0134	0.0172^c^	0.0284	0.0219	0.0251^b^	0.0333	0.0252	0.0292^b^
T_5_	0.0241	0.0138	0.0190^a^	0.0249	0.0146	0.0198^b^	0.0312	0.0230	0.0271^b^	0.0321	0.0253	0.0287^b^
T_6_	0.0270	0.0149	0.0210^a^	0.0278	0.0163	0.0221^a^	0.0380	0.0256	0.0318^a^	0.0343	0.0284	0.0313^a^
Mean	0.0186^a^	0.0113^b^		0.0202^a^	0.0125^b^		0.0291^a^	0.0222^b^		0.0325^a^	0.0248^b^	
EM SA EM×SA SA×EM	**	**		**			**			**		
	**	**		**			**			**		
	**	**		**			ns			**		
	**	**		**			ns			**		

*SRI, system of rice intensification; CM, conventional management; EM, establishment method, SA, soil amendment; EM×SA, Interaction of establishment methods at same level of soil amendments; SA×EM, Interaction of soil amendments at same level of establishment methods. T_1_, No amendment; T_2_, 5 tonnes biochar ha^-1^; T_3_, 10 kg polymerha^-1^; T_4_, 5 tonnes biochar ha^-1^ + 10 kg polymerha^-1^; T_5_, 10 tonnes biochar ha^-1^; T_6_, 10 tonnes biochar ha^-1^ + 10 kg polymerha^-1^.

Different letters within the same column indicate significant differences between treatments, as determined by Tukey’s test with *p<* 0.05.

ns and **denotes not significant and significant differences at the 5% level, respectively.

Similarly, root diameter (RD) and root volume (RV) were significantly affected by planting methods and SA (**Table 7**). In general, SRI recorded 45.1% and 47.8% greater RD and RV, respectively, than in CM. Among the soil amendments, T_6_ had the highest average RD and RV (1.10 cm, 27.55 cc hill^-1^), and the control evinced the lowest RD and RV. Similarly, in2020 and 2021, the root diameter and volume in the T6 treatment increased by 2.06 and 1.48 and 1.42 and 1.20, respectively, compared to the control treatment. Root morphology improved in the SRI in both years, demonstrating that SRI outperformed CM. RLD and RWD exhibited a 1.31-fold and 1.62-fold increase in SRI compared to CM.

**Table 7:** Effect of establishment methods and soil amendments on root diameter (RD) and root volume (RV) of rice.

Treatments	Root diameter (cm)						Root volume (cm^3^ hill^-1^)					
	2020		Mean	2021		Mean	2020		Mean	2021		Mean
	Establishment methods			Establishment methods			Establishment methods			Establishment methods		
	SRI*	CM		SRI	CM		SRI	CM		SRI	CM	
T_1_	0.71	0.55	0.63^e^	0.84	0.61	0.73^e^	21.30	12.40	16.85^c^	29.48	20.47	24.98^c^
T_2_	0.75	0.59	0.67^e^	0.97	0.71	0.84^d^	23.93	13.13	18.53^bc^	30.03	22.21	26.12^b^
T_3_	0.89	0.62	0.76^d^	1.04	0.65	0.85^d^	24.30	14.23	19.27^bc^	27.33	23.95	25.64^c^
T_4_	1.03	0.69	0.86^c^	1.12	0.78	0.95^c^	27.07	14.67	20.87^b^	30.08	24.00	27.04^b^
T_5_	1.17	0.76	0.96^b^	1.20	0.82	1.01^b^	27.67	15.40	21.53^b^	30.18	24.07	27.13^b^
T_6_	1.36	0.83	1.10^a^	1.31	0.92	1.11^a^	32.43	17.53	24.98^a^	33.83	26.42	30.13^a^
Mean	0.98^a^	0.67^b^		1.08^a^	0.75^b^		26.12^a^	14.56b		30.16^a^	23.52^b^	
EM SA EM×SA SA×EM	**			**			**			**		
	**			**			**			**		
	**			**			ns			**		
	**			**			ns			**		

*SRI, system of rice intensification; CM, conventional management; EM, establishment method; SA, soil amendment; EM×SA, Interaction of establishment methods at same level of soil amendments; SA×EM, Interaction of soil amendments at same level of establishment methods. T_1_, No amendment; T_2_, 5 tonnes biochar ha^-1^; T_3_, 10 kg polymerha^-1^; T_4_, 5 tonnes biochar ha^-1^ + 10 kg polymerha^-1^; T_5_, 10 tonnes biochar ha^-1^; T_6_, 10 tonnes biochar ha^-1^ + 10 kg polymerha^-1^.

Different letters within the same column indicate significant differences between treatments, as determined by Tukey’s test with *p<* 0.05.

ns and **denotes not significant and significant differences at the 5% level, respectively.

### 3.5 Water productivity and water savings

**Figure 5:**
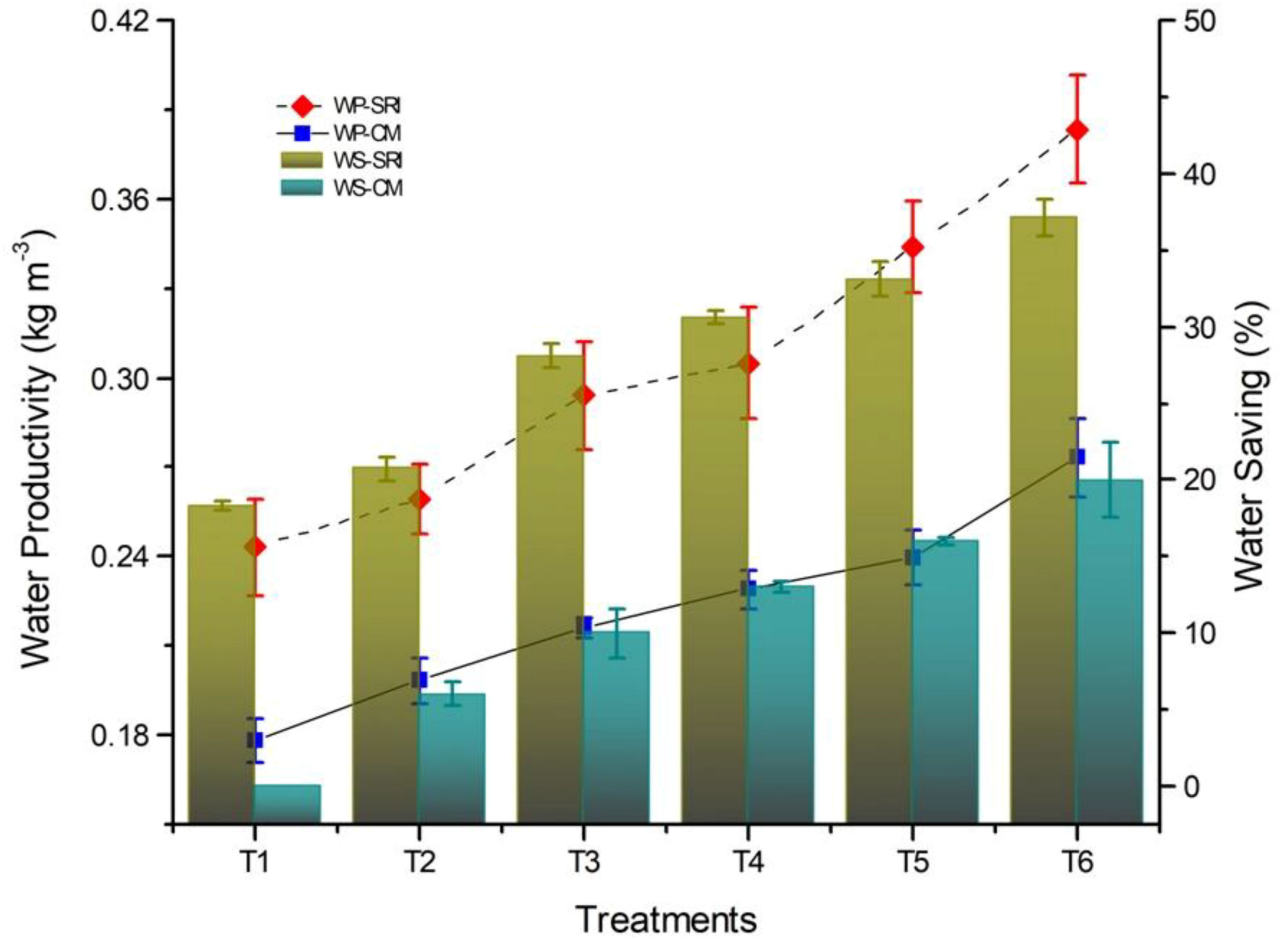
Pooled water productivity (WP) and water saving (WS) among various establishment methods and amendments. Bars indicate standard error from mean P value< 0.05. SRI, System of rice intensification; CM, Conventional management; T_1_, No amendment; T_2_, 5 tonnes biochar ha^-1^; T_3_, 10 kg polymerha^-1^; T_4_, 5 tonnes biochar ha^-1^+ 10 kg polymerha^-1^; T_5_, 10 tonnes biochar ha^-1^; T_6_, 10 tonnes biochar ha^-1^+ 10 kg polymerha^-1^.

The EM and SA significantly impacted water productivity (WP) and water saving (WS) (**Figure 5**). The results indicated that SRI saved 28.2% of irrigation water compared toCM across two years. Among the soil amendments, T_6_ produced the highest average WS (29.45%) over the control. Similarly, for both years, the average WP in SRI was 46.5% higher than in the CM. Similarly, T_6_ had the highest average WP of 0.34 kg m^-3^. In 2020, no significant interaction was recorded between EM and SA on WS and WP. However, in 2021, SRI×T_6_ showed the highest values (41.81%, 0.44 kg m^-3^, respectively) of WS and WP, owing to the beneficial effects of biochar and polymer on soil water retention.

### 3.6 Principal component analysis and correlation matrix

**Figure 6:**
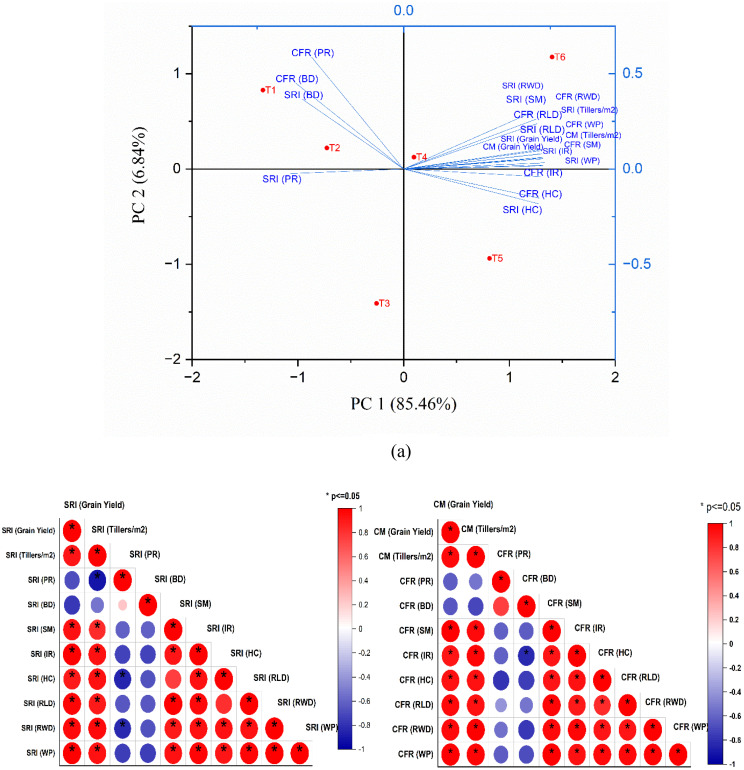
**(a)** Biplot Principal Component Analysis (PCA) plot, **(b)** Heatmap correlation analysis diagram of treatment methods and observed parameters for rice cultivation.

**Figures 6a, b** display the PCA and correlation matrix, encompassing data on treatment techniques and analyzed soil and crop parameters. **Figure 6a** illustrates that PC1 (x-axis) accounts for 85.46% of the variation in the data, whereas PC2 (y-axis) accounts for 6.84% of the variance. Consequently, only two main components, PC1 and PC2, were used in the PCA analysis. The principal component analysis revealed that PC1 exhibited dominant loadings from variables including SRI (RWD), SRI (SM), CFR (continuous flooded rice) (RWD), SRI (Tillers m^-^²), CFR (WP), and CM (Grain Yield), all of which displayed the longest vector projections along the PC1 axis. This pattern indicates that PC1 primarily encapsulated the variance associated with productivity-linked agronomic traits, reflecting their strong interdependence and contribution to overall system performance. In contrast, PC2 accounted for only 6.84% of the total variability, with notable but weaker loadings from CFR (PR), CFR (BD), and SRI (BD), suggesting that these parameters exerted comparatively minor influence on the overall variance structure. The PCA biplot (**Figure 6a**) illustrates the directional influence and magnitude of each variable through the orientation and length of the blue vectors relative to the principal components.

Furthermore,the PCA analysis biplot depicted in **Figure 6a** indicates that among all treatment approaches, T6 (10 tonnes of biochar ha^-1^+ 10 kg of polymer ha^-1^) had the most significant influence, demonstrating a positive connection with PC1. Additionally, several metrics, including SRI (HC), CFR (IR), and CFR (HC), exhibited a moderate positive correlation with the T5 treatment approach (10 tonnes of biochar ha^-1^). CFR (PR), CFR (BD), and SRI (BD) had a significant negative correlation with treatment T1 (no modification). Ultimately, SRI (PR) had a neutral impact on any treatment modality. The biplot PCA analysis indicated that the SRI cultivation approach, utilising 10 tonnes of biochar and 10 kg of polymer amendment, yields superior outcomes compared to alternative methods. The heatmap correlation diagram presented in **Figure 6b** corroborates that in the SRI cultivation methodology, the treatment techniques exhibit a robust positive association with grain yield, tiller, SM, IR, HC, RLD, RWP, and WP, while demonstrating a high link with SRI PR and BD. These results align with the data obtained from PCA analysis, indicating that SRI BD and PR were negatively linked with PC1. Similarly, in the CFR approach, the treatment procedures had a positive correlation with grain yield, IR, HC, RLD, RWP, and WP, while demonstrating a negative correlation with PR and BD.

The PCA loading of each variable with respect to PC1 and PC2 is shown in **Supplementary Table 2**. The PCA outcomes demonstrate that agronomic performance and yield-related variables distinctly segregate along the two principal components, PC1 and PC2, which together account for 92.3% of the total variance.PC1 alone explains 85.46%, serving as the dominant axis of differentiation among treatments. Variables associated with WP [SRI (WP), CFR (WP)], grain yield [SRI (Grain Yield), CM (Grain Yield)], and tiller density [SRI (Tillers m^-2^), CM (Tillers m^-2^)] exhibit the highest loadings, ranging approximately from 5.1% to 5.8%, indicating their strong collective contribution to PC1. This implies that PC1 primarily represents the combined influence of yield efficiency and growth potential, where positively clustered loadings reflect mutual enhancement among these traits, thereby emphasizing their synergistic role in determining crop performance.

Additional variables such as SRI (RWD), CFR (RWD), SRI (IR), and CFR (IR) also exhibit moderate to high positive associations with PC1, reinforcing the centrality of grain and WP traits in shaping the primary axis. PC2, accounting for 6.84% of the variability, delineates a secondary but meaningful dimension, dominated by CFR (PR) (34.47%), CFR (BD) (20.35%), and SRI (BD) (13.42%), which correspond to biomass density and post-rainfall responses. This pattern suggests that PC2 captures structural or environmental variability, distinct from the yield-oriented variance encapsulated in PC1. Moderate loadings from SRI (SM) and CFR (SM) (ranging between 8.8% and 0.9%) further highlight phenological and soil-moisture interactions that complement productivity traits. Collectively, this two-axis differentiation underscores the multifactorial architecture of crop performance, wherein PC1 reflects integrated physiological and yield attributes, whilePC2 isolates environmental and biomass-related influences. This separation clarifies the relative contribution of each agronomic parameter and offers a strategic basis for optimizing management practices, aligning yield enhancement with resource efficiency and environmental adaptability.

## 4 Discussion

In the present study, significant improvement in soil physicochemical properties was observed in the combined application of biochar and polymer treatments compared to the sole application. Co application of biochar and polymer (T_6_: 10 tonnes biochar ha^-1^ + 10 kg polymerha^-1^) increased the soil moisture (1.2 times) and reduced the soil PR (1.15 times), and improved the soil water movement (IR by 1.8 and HC by 1.6 times) across the year as compared to the T_1_ (no amendment) in SRI practice. The SRI system increased SM by 6.6%, increased IR by 5.26%, increased HC by 3.08%, and reduced PR and BD over the CM. These findings indicate that the SRI method effectively manages soil, plants, and water in rice cultivation. Previous studies have also shown that the SRI significantly impacts water movement and soil compaction (Gathorne-Hardy et al., 2016).Studies suggest that porous nature (**Figure 2**) and presence of aromatic surface functional groups of biochar (**Figure 3**) contribute to efficient water distribution, enhanced nutrient availability, improve microbial diversity and collectively promote root development (Saini et al., 2025; Sharma et al., 2025b; Bolan et al., 2024).

The results are in line with the findings of Yu et al. (2013) and Chen et al. (2024). The O/C ratio (**Figure 1**) for the biochar suggests that biochar possesses a moderate level of aromaticity (resistance to microbial degradation) and stability in the soil, making it very appropriate for improving soil physicochemical qualities (Lehmann and Joseph, 2024).Finer biochar particles dissolve in irrigation water, increasing the electrolyte concentration in the soil solution as evidenced by significantly higher electrical conductivity (**Supplementary Table 3**), further decreasing the zeta potential. Meanwhile, the dissolved polymer assumes a coiled form rather than long linear chains (Barvenik, 1994), entrapping soil and biochar particles to create water-stable aggregates, contributing to increased water retention and higher IR and HC under high electrolyte concentration. The increased activity of the polymer is attributed to the presence of electrolytes (Yu et al., 2003). Additionally, the released electrolytes decreased the viscosity of the polymer and increased infiltration (Abrol et al., 2016). Thus, biochar was found to regulate the efficacy of the polymer. Our findings aligned with previous studies that demonstrated that the electrolytes are essential for the beneficial effect of polymer on soil water movement and that using biochar as an electrolyte source resulted in significantly higher EC values due to the release of cations in the soil solution (Abrol et al., 2013; Abrol et al., 2016).

Biochar mixed with polymer leads to a significant change in effective tillers density and rice yield, compared to sole application and control treatments (**Table 3**). Our findings indicate that the maximum tillers m^-2^ was obtained with the T_6_ treatment under the SRI establishment practice. Several studies confirm our results (Demeyer et al., 2001; Bagheri et al., 2021). The highest rice yield was achieved under the joint application of biochar and polymer (T_6_), resulting in a 22.4% increase over the control treatment under SRI (**Figure 4**). This difference has been attributed to the wider spacing between plants in SRI, which promotes better plant and root growth and produces more tillers than does the conventional method (Thakur et al., 2013; Bagheri et al., 2021). The yield increase was correlated with aerobic conditions in SRI relative to CM, which influenced soil physicochemical and biological properties and created a favorable environment for plant and root growth. Previous research from eight countries has also reported that SRI increased rice yields by up to 47% while using 40% less water than conventional methods (Sato and Uphoff, 2007; Africare, 2010). Our results demonstrate the positive effects of biochar and polymer amendments on rice yield. Furthermore, a substantial increase in yield was obtained when biochar was coupled with polymer as compared to their sole application. These findings suggest that the synergistic interaction between biochar and polymer improves soil structure and enhances nutrient availability during plant growth (Abdeen et al., 2023; Rafique et al., 2025). An increased in crop yield could be linked to biochar’s beneficial influence on soil physical characteristics and microbial activity (Sharma et al., 2025a; Bolan et al., 2024). Laird et al. (2010) also observed an improvement in soil physical properties and available nutrients with biochar incorporation in soil.

**Figure 7:**
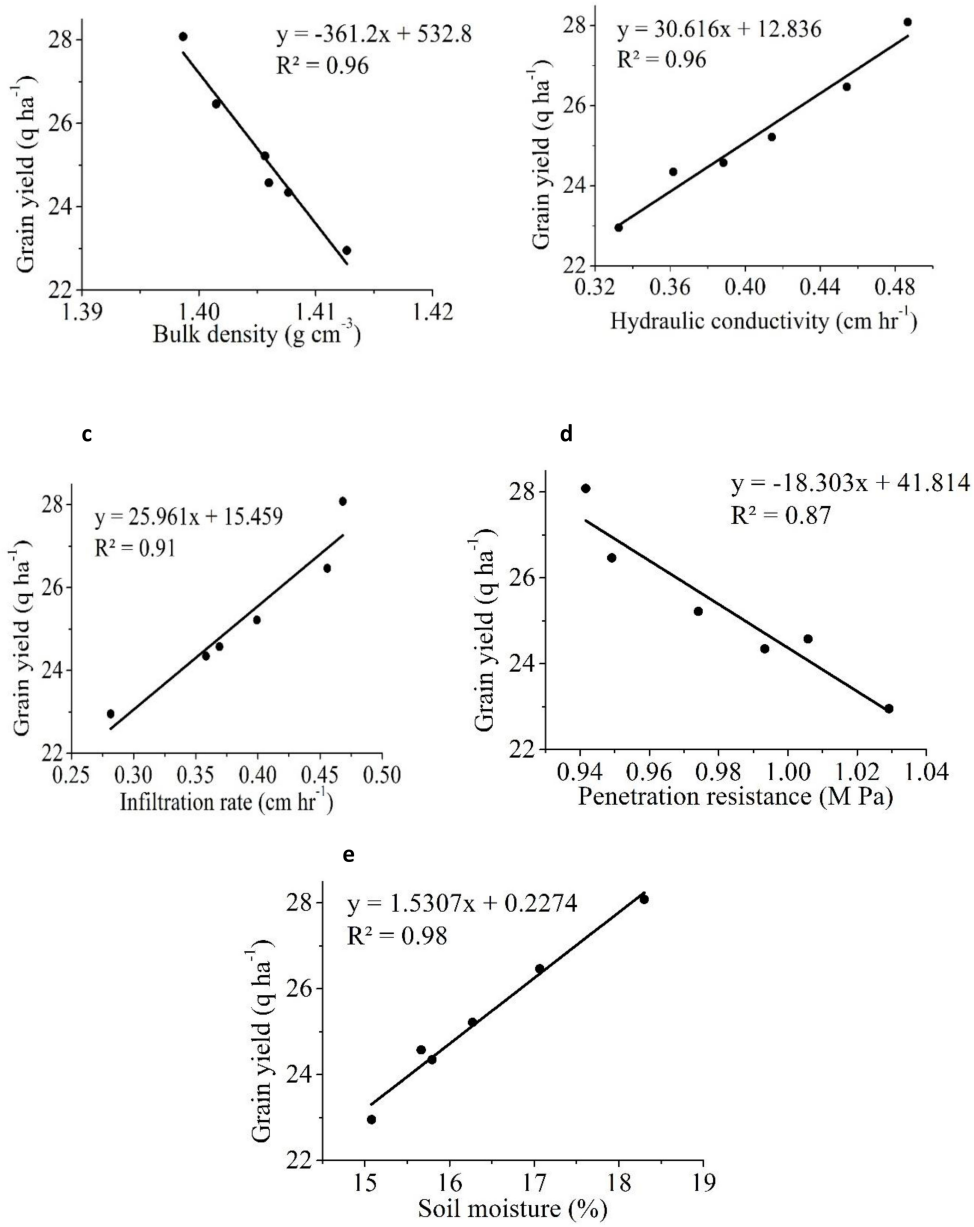
The linear relationship between rice grain yield and different soil physical properties **(a)** bulk density (BD) **(b)** hydraulic conductivity (HC) **(c)** infiltration rate (IR) **(d)** penetration resistance (PR) **(e)** soil moisture (SM).

Conventional flooding conditions have an adverse impact on plant root growth because of the toxic effects of ferrous iron and manganese, which can negatively affect rice root growth (Olaleye et al., 2001). The study found that the SRI system significantly improved crop root growth by improving the soil rhizosphere environment by alternate wetting and drying, which increased oxygen supply to rice roots (Stoop et al., 2002; Ishfaq et al., 2020; Mboyerwa et al., 2022). In addition, an increase in rice yield under SRI was correlated with rapid root growth due to relatively conducive aerobic soil and increased microbial and enzymatic activity, which might have improved photosynthetic rate and, hence, crop yield (Yang et al., 2004; Zhang et al., 2009; Wang et al., 2011; Li et al., 2016). The presence of carbon, phosphorus, nitrogen, and potassium (**Table 2**) in rice husk biochar provides a conducive soil environment for root growth. Phosphorus is a vital nutrient necessary for root development and energy transmission in crops, whereas nitrogen and potassium are crucial for leaf growth, water retention, and stress management, respectively (Lehmann and Joseph, 2024). The incorporation of porous and microporous biochar into the soil apparently improves its water retention and nutrient retention capabilities (Yu et al., 2013).The significant increase in rice yield and root morphology was attributable to the positive correlation between soil physical conditions (SM, IR, and HC) and the negative correlation between BD and PR (**Figure 7**).

**Figure 8:**
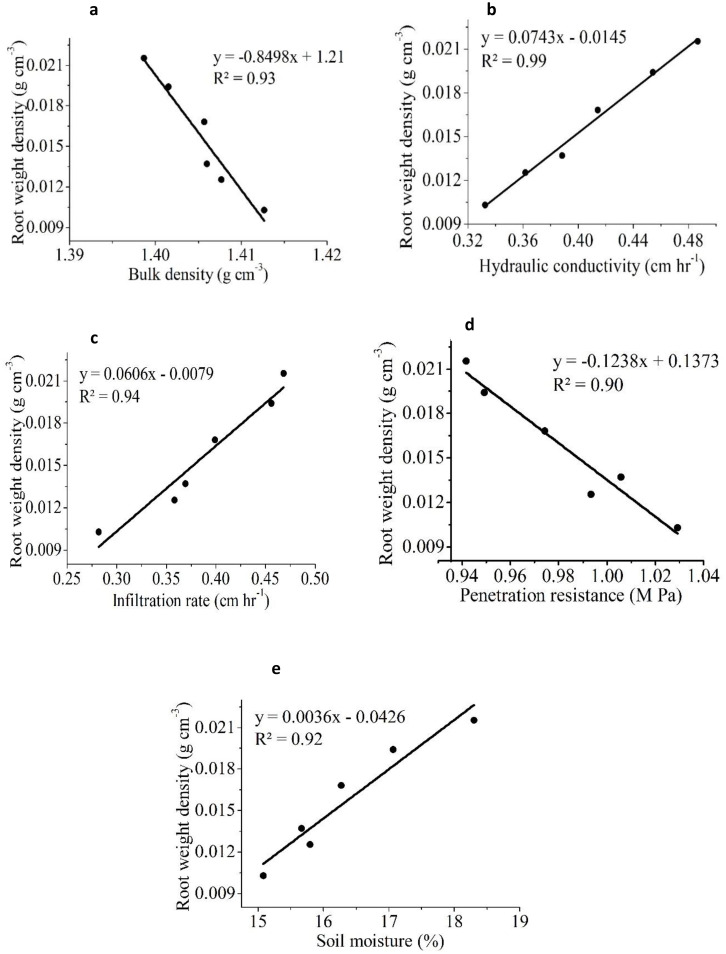
The linear relationship between root length density (RLD) and different soil physical properties **(a)** bulk density (bd) **(b)** hydraulic conductivity (BC) **(c)** infiltration rate (IR) **(d)** penetration resistance (PR) **(e)** soil moisture (SM).

**Figure 9:**
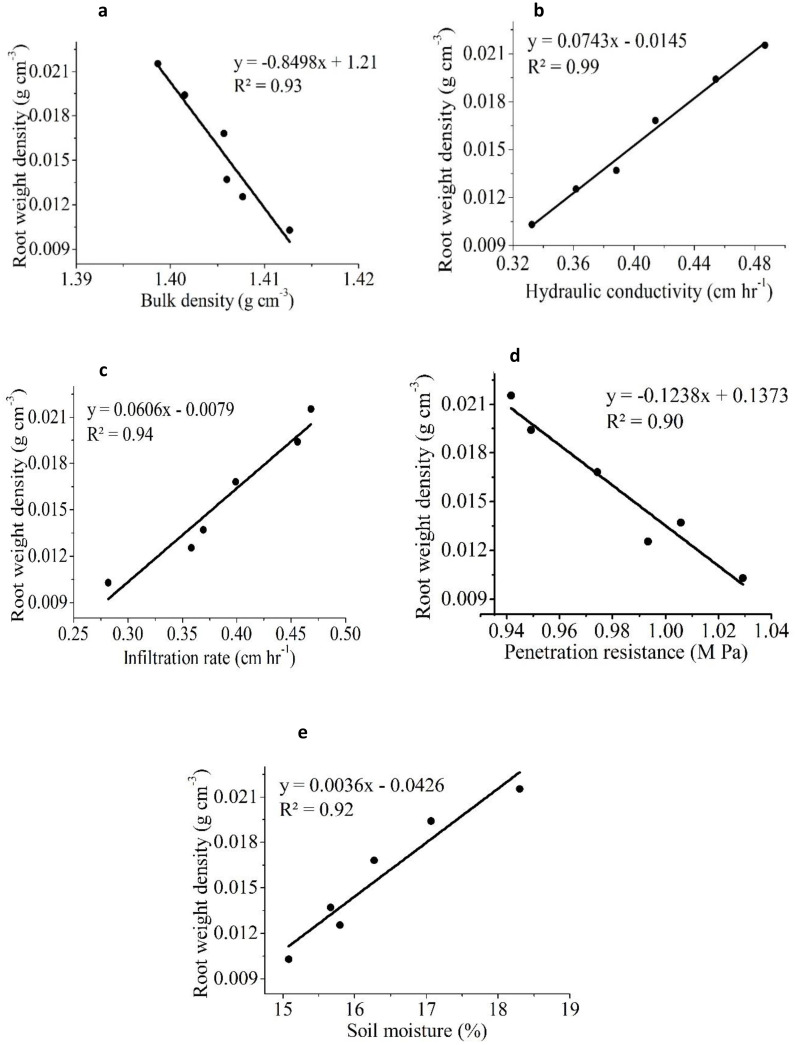
The linear relationship between root weight density (RWD) and different soil physical properties **(a)** bulk density (BD) **(b)** hydraulic conductivity (HC) **(c)** infiltration rate (IR) **(d)** penetration resistance (PR) **(e)** soil moisture (SM).

**Figure 10:**
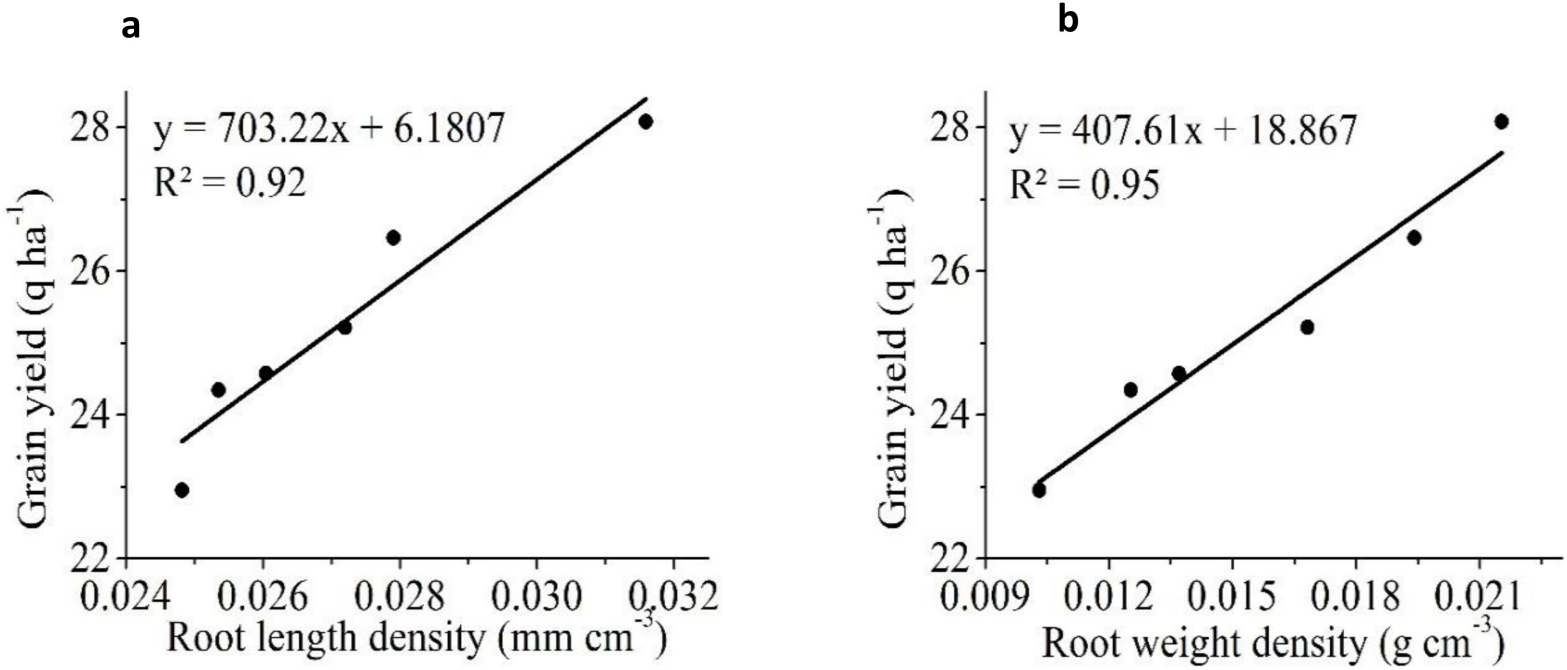
The linear relationship between rice grain yield **(a)** root length density (RLD) **(b)** root weight density (RWD).

Similar results in a previous study reportedly showed a significant improvement in rice yield due to a positive change in soil properties in SRI that used less water than CM (Stoop et al., 2002; Pascual and Wang, 2016). **Figure 7** shows that rice yield was significantly and positively associated with IR (R^2^ = 0.91), HC (R^2^ = 0.93), and SM (R^2^ = 0.98), but was negatively correlated with BD (R^2^ = 0.96) and PR (R^2^ = 0.97). The results showed that BD had the strongest negative correlation with yield, followed by PR. Several researchers have demonstrated that increased soil compaction reduces crop yield, possibly due to poor aeration and nutrient availability (Sharma et al., 2021; Tripathi et al., 2005). Similarly, RLD was positively correlated with IR, HC, and SM but negatively correlated with BD and PR (**Figure 8**). RWD had strong positive relationships with IR, HC, and SM (**Figure 9**). A significant correlation was found between the RLD (R^2^ = 0.92), and RWD (R^2^ = 0.95), which improved soil physical properties, favoring a soil environment that promoted higher crop yield (**Figure 10**).

Furthermore, the strong positive relationships of grain yield with RLD and RWD are consistent with previous findings (Blanco et al., 2013). In addition, PCA analysis demonstrated that, except BD and PR, all other parameters were influenced by the kind of amendment, regardless of the culture mode employed. Furthermore, several metrics, including SRI (RWD), SRI (SM), SRI (RLD), SRI (grain yield), SRI (Tillersm^-2^), and SRI (WD), exhibited a robust correlation with the T6 treatment, suggesting that optimal outcomes in rice cultivation may be attained by this strategy. The PCA analysis further validated that the T_6_ amendment technique yielded the most favourable outcomes compared to all other amendment methods.

Among all the treatments, SRI establishment practice with biochar and polymer (T_6_) showed the highest WS and WP values (41.81%, 0.44 kg m^-3^, respectively). The effect of biochar and polymer could be beneficial on soil water retention (Abrol et al., 2013, 2016; Alkhasha et al., 2018). Our findings suggest that it can save 39.45% of the water if the conventional planting method is replaced with SRI×T_6._ The results of our study suggest that biochar with polymer plays a crucial role in the improvements of soil hydraulic functions and root growth, thereby facilitating an increase in rice grain yield and WP.

## 5 Conclusion

This study revealed that soil additives (biochar and polymer) and rice establishment methods (SRI, traditional) substantially influenced soil hydraulic properties, water retention, root development, and rice production. A 15% increase in rice production attained using the SRI compared to CM illustrates a soil condition that promotes root development, hence enhancing the availability of water and nutrients. WP increased by 1.7 times, and irrigation water depth was reduced by 39.45%, confirming the synergistic good effects of biochar and polymer under the SRI. Therefore, implementation of SRI×T_6_ (10 tonnes of biochar ha^-1^ and 10 kg of polymer ha^-1^) is a potential strategy for attaining increased basmati rice yields with reduced irrigation water usage. Nevertheless, the future research should more focus on understanding the long-term impacts of biochar–polymer integration on soil microbial dynamics, nutrient cycling, and carbon sequestration across diverse soil types and climatic conditions. Additionally, further field experiments are required to observe the long-term impacts of biochar–polymer integration on different soil properties and agricultural productivity across various agroecosystems.

## Data Availability

The datasets presented in this study can be found in online repositories. The names of the repository/repositories and accession number(s) can be found in the article/**Supplementary Material**.
