# Supplementary material for: Biochar and anionic polyacrylamide modulated soil hydraulic functions catalyze water saving, root development and yield of basmati rice
Source: Front Plant Sci. 2025 Dec 11;16:1660325. doi: 10.3389/fpls.2025.1660325 (PMC12739761; doi:10.3389/fpls.2025.1660325)
Supplement: Supplementary file 1 [file Table1.docx]

**Table 1**: Crop consumptive use coefficient (K_c_) for calculating irrigation requirement.

| Percent of Crop Growing Season | | Consumptive use (Evapo-transpiration) coefficients, k, to be Multiplied by Class A Pan Evaporation or Calculated E | | | | | | | | |
| --- | --- | --- | --- | --- | --- | --- | --- | --- | --- | --- |
|  |  | Group A | | Group B | Group C | Group D | Group E | Group F | Group G | Rice |
| 0 | | | 0.20 | 0.15 | 0.12 | 0.08 | 0.90 | 0.60 | 0.50 | 0.80 |
| 5 | | | 0.20 | 0.15 | 0.12 | 0.08 | 0.90 | 0.60 | 0.55 | 0.90 |
| 10 | | | 0.36 | 0.27 | 0.22 | 0.15 | 0.90 | 0.60 | 0.60 | 0.95 |
| 15 | | | 0.50 | 0.38 | 0.30 | 0.19 | 0.90 | 0.60 | 0.65 | 1.00 |
| 20 | | | 0.64 | 0.48 | 0.38 | 0.27 | 0.90 | 0.60 | 0.70 | 1.05 |
| 25 | | | 0.75 | 0.56 | 0.45 | 0.33 | 0.90 | 0.60 | 0.75 | 1.10 |
| 30 | | | 0.84 | 0.63 | 0.50 | 0.40 | 0.90 | 0.60 | 0.80 | 1.14 |
| 35 | | | 0.92 | 0.69 | 0.55 | 0.46 | 0.90 | 0.60 | 0.86 | 1.17 |
| 40 | | | 0.97 | 0.73 | 0.58 | 0.52 | 0.90 | 0.60 | 0.90 | 1.21 |
| 45 | | | 0.99 | 0.74 | 0.60 | 0.58 | 0.90 | 0.60 | 0.95 | 1.25 |
| 50 | | | 1.00 | 0.75 | 0.60 | 0.65 | 0.90 | 0.60 | 1.00 | 1.30 |
| 55 | | | 1.00 | 0.75 | 0.60 | 0.71 | 0.90 | 0.60 | 1.00 | 1.30 |
| 60 | | | 0.99 | 0.74 | 0.60 | 0.77 | 0.90 | 0.60 | 1.00 | 1.30 |
| 65 | | | 0.96 | 0.72 | 0.58 | 0.82 | 0.90 | 0.60 | 0.95 | 1.25 |
| 70 | | | 0.91 | 0.68 | 0.55 | 0.88 | 0.90 | 0.60 | 0.90 | 1.20 |
| 75 | | | 0.85 | 0.64 | 0.51 | 0.90 | 0.90 | 0.60 | 0.85 | 1.15 |
| 80 | | | 0.75 | 0.56 | 0.45 | 0.90 | 0.90 | 0.60 | 0.80 | 1.10 |
| 85 | | | 0.60 | 0.45 | 0.36 | 0.80 | 0.90 | 0.60 | 0.75 | 1.00 |
| 90 | | | 0.46 | 0.35 | 0.28 | 0.70 | 0.90 | 0.60 | 0.70 | 0.90 |
| 95 | | | 0.28 | 0.21 | 0.17 | 0.60 | 0.90 | 0.60 | 0.55 | 0.80 |
| 100 | 0.20 | | | 0.20 | 0.17 | 0.20 | 0.90 | 0.60 | 0.50 | 0.20 |
