# Supplementary material for: Biochar and anionic polyacrylamide modulated soil hydraulic functions catalyze water saving, root development and yield of basmati rice
Source: Front Plant Sci. 2025 Dec 11;16:1660325. doi: 10.3389/fpls.2025.1660325 (PMC12739761; doi:10.3389/fpls.2025.1660325)
Supplement: Supplementary file 2 [file Table2.docx]

**Table 2**: Summary of the PCA loading of each variable with respect to PC1 and PC2.

| **Variable** | **PC1 Loading** | **PC1 (%)** | **PC2 Loading** | **PC2 (%)** |
| --- | --- | --- | --- | --- |
| SRI (Grain Yield) | 0.23647 | 5.59 | 0.06114 | 0.37 |
| CM (Grain Yield) | 0.23584 | 5.56 | 0.08029 | 0.64 |
| SRI (Tillers/m2) | 0.23112 | 5.34 | 0.07807 | 0.61 |
| CM (Tillers/m2) | 0.23652 | 5.59 | 0.10443 | 1.09 |
| SRI (PR) | -0.19336 | 3.74 | -0.02362 | 0.06 |
| CFR (PR) | -0.15737 | 2.48 | 0.58713 | 34.47 |
| SRI (BD) | -0.17301 | 2.99 | 0.36633 | 13.42 |
| CFR (BD) | -0.18432 | 3.40 | 0.45116 | 20.35 |
| SRI (SM) | 0.21902 | 4.80 | 0.29693 | 8.82 |
| CFR (SM) | 0.23591 | 5.57 | 0.09705 | 0.94 |
| SRI (IR) | 0.23673 | 5.60 | 0.02064 | 0.04 |
| CFR (IR) | 0.23563 | 5.55 | -0.04102 | 0.17 |
| SRI (HC) | 0.23060 | 5.32 | -0.18243 | 3.33 |
| CFR (HC) | 0.23125 | 5.35 | -0.15267 | 2.33 |
| SRI (RLD) | 0.22835 | 5.21 | 0.23859 | 5.69 |
| CFR (RLD) | 0.22670 | 5.14 | 0.26261 | 6.90 |
| SRI (RWD) | 0.23763 | 5.65 | 0.01639 | 0.03 |
| CFR (RWD) | 0.23739 | 5.64 | 0.05369 | 0.29 |
| SRI (WP) | 0.24014 | 5.77 | 0.03265 | 0.11 |
| CFR (WP) | 0.23913 | 5.72 | 0.05835 | 0.34 |
