# Supplementary material for: Biochar and anionic polyacrylamide modulated soil hydraulic functions catalyze water saving, root development and yield of basmati rice
Source: Front Plant Sci. 2025 Dec 11;16:1660325. doi: 10.3389/fpls.2025.1660325 (PMC12739761; doi:10.3389/fpls.2025.1660325)
Supplement: Supplementary file 3 [file Table3.docx]

**Table 3**: ANOVA table of soil reaction (pH) and electrical conductivty.

| LSD (5%) |  | |  |
| --- | --- | --- | --- |
|  | pH |  | Electrical conductivty (dSm^-1^) |
| EM | NS |  | NS |
| SA | 0.01 |  | 0.008 |
| EM×SA | NS |  | NS |
